# “Telephone consultation for medical emergencies” – development, implementation and evaluation of a course in identifying and handling medical emergencies via telephone for 5^th^ year medical students

**DOI:** 10.3205/zma001459

**Published:** 2021-03-15

**Authors:** Beate Gabriele Brem, Kai Philipp Schnabel, Ulrich Woermann, Roman Hari, Anina Pless

**Affiliations:** 1 University of Bern, Institute for Medical Education, Bern, Switzerland; 2 University of Bern, Institute of Primary Health Care, Bern, Switzerland

**Keywords:** telephone consultation, simulated patients, communication skills training

## Abstract

**Objectives: **Advising patients seeking medical guidance while communicating with them via telephone is a highly relevant skill in clinical daily life. However, telephone consultations differ from face-to-face interactions: clinical examination is nearly impossible and visual signals cannot be observed. Thus, telephone consultations require specific skills training. This article describes the development, implementation and evaluation of a course, “Telephone Consultation for Medical Emergencies”, for 5^th^ year medical students at the University of Bern, Switzerland.

**Methods: **Following the evidence in the literature for telephone consultations, we developed guidelines for effective communication via telephone. After self-study of preparatory material, learners engaged in telephone consultations with simulated patients (SP) at the simulation center. They received multi-dimensional feedback regarding the encounter.

**Results: **The course was successfully implemented in 2012. Evaluations showed the course to be well-received by students. In a survey, students agreed that they had learned many new skills and that they considered this learning as being important in their future employment. They felt that the SP feedback was helpful and that being observed by peer-students during the encounter or filling in a checklist while observing peer-students in other encounters added to their learning. During the debriefing of the simulation with a clinical expert, students judged the scenarios as realistic and relevant, praised the SP performances and identified that the most instructive aspect of the training was the opportunity to practice and to get feedback.

**Conclusion: **Telephone consultations require specific skills that should be trained. The current Covid-19 pandemic and the recommendations of government institutions for patients to contact healthcare professionals primarily via telephone stress the importance of adequately training these skills. In this publication we describe a feasible and viable format for implementing this process.

## Zusammenfassung

**Zielsetzung: **Patienten und Patientinnen, die medizinischen Rat suchen, mittels eines Telefongespräches zu beraten, ist eine sehr wichtige Fähigkeit in der täglichen klinischen Routine. Allerdings unterscheidet sich die Kommunikation über das Telefon von einer persönlichen Konsultation: eine klinische Untersuchung ist nahezu unmöglich und es können keine visuellen Signale beobachtet werden. Daher ist ein gesondertes Training der telefonischen Beratung erforderlich.

Dieser Beitrag beschreibt die Entwicklung, Durchführung und Evaluation eines Kurses für „Telefonische Notfallkommunikation“ für Medizinstudierende im 5. Studienjahr an der Universität Bern, Schweiz.

**Methoden:** Unter Berücksichtigung der Evidenz bezüglich telefonischer Beratungen in der Literatur haben wir Richtlinien für eine wirksame telefonische Kommunikation entwickelt. Nach dem Selbststudium vorbereitender Materialien, üben die Studierenden die telefonische Beratung mit Simulationspatienten (SP) im Simulationszentrum. Sie erhalten bezüglich der Simulation multi-dimensionales Feedback.

**Ergebnisse: **Der Kurs wurde erstmals 2012 erfolgreich durchgeführt. Die Evaluationen zeigten, dass der Kurs von den Studierenden positiv aufgenommen wurde. In einer Umfrage stimmten die Studierenden zu, dass sie viele neue Fertigkeiten erlernt hätten und dass sie das Gelernte für ihre zukünftige Arbeit als wichtig erachteten. Sie hatten das Gefühl, dass das Feedback der SP für sie hilfreich war und dass die Beobachtung während der Simulation durch einen Peer-Studierenden oder das Ausfüllen einer Checkliste während der Beobachtung eines Peer-Studierenden in anderen Simulationen zusätzlich zu ihrem Lerneffekt beitrugen. Im Rahmen von Debriefings von Simulationen mit klinischen Experten beurteilten die Studierenden die Fallbeispiele als realistisch und relevant, lobten die Darstellung der Rollen durch die SP und benannten die Möglichkeit zum Üben und das Erhalten von Feedback als die lehrreichsten Elemente des Kurses.

**Schlussfolgerung: **Die telefonische Beratung erfordert spezielle Fähigkeiten, die gesondert trainiert werden sollten. Die derzeitige Covid-19 Pandemie und die Empfehlungen der Behörden, dass Patienten Gesundheitspersonal bevorzugt über das Telefon kontaktieren sollten, betont die Wichtigkeit eines angemessenen Trainings dieser Fähigkeiten. In dieser Publikation beschreiben wir ein machbares und brauchbares Format für einen solches Training.

## 1. Introduction

Means of communication have changed significantly in the last century. While in the past a patient seeking a doctor’s advice did so during a face-to-face consultation, today first contact via telephone is a viable alternative. Telephone consultations are offered in many countries including the United States of America (USA) [1], the United Kingdom [2], Australia [3] and Switzerland [4]. The importance of telephone consultations increased considerably during the COVID-19 pandemic in the spring of 2020. For example, the Federal Office of Public Health in Switzerland instructed patients with symptoms of an infection to contact health professionals by telephone before seeing a doctor in person [5]. 

Telephone communication differs significantly from face-to-face interaction. In a telephone consultation it is nearly impossible for the physician to do a physical exam. Visual clinical signs like paleness or sweating go unrecognized and it is not possible to pick up visual signs of communication [6]. Telephone consultations require high verbal cue sensitivity [7]. A long pause between words may indicate a patient with respiratory distress or rapid speech may be a symptom of an anxious patient. In telephone consultations, it is important to take a focused history, to be aware of situations in which telephone consultations are inappropriate, and to take care of adequate documentation. Physicians need to guide and counsel patients about home management including self-monitoring and follow-up arrangements. They have to know how to negotiate a plan with a patient and assess its feasibility [7]. In 2007 Derks et al. generated a tool for quality measurement of telephone consultations containing 17 items characterizing effective calls. They organized the items according to 4 phases: **R**eason for calling; **I**nformation to be gathered; **C**onclusion and care advice given; and **E**valuation of the call. The tool is named “RICE” after its acronym [8], [9]. Since the start of the COVID-19 pandemic, additional various guidelines and tips on how to communicate efficiently by phone have been published [10], [11], [12].

Little is known about effective means to teach telephone consultation skills [13]. A qualitative study conducted by Eppich et al. explored how telephone conversations between physicians should be taught [14]. Telephone conversations between physicians occur for example, when doctors call a specialist for advice. Eppich et al. interviewed young residents with regard to their needs in learning telephone conversations. The residents identified feedback and explicit teaching strategies as most important for their learning. 

The teaching strategies identified to be important were [14]:

- providing brief background information about a given issue before giving specific advice how to deal with it (i.e. if it is difficult to follow the logic of the presentation of a patient case because the adequate structure is missing, the instructor should give some information about the correct structure of patient presentations in general before advising the learner to improve the structure of his/her presentation by following a certain order when presenting the facts).
- explaining not only facts, but also the rationale to encourage learning of the “thought-process” (i.e. if the learner presented irrelevant information while paradoxically leaving out critical details, it is important for him/her to understand how irrelevant information can distract the focus from the important things and why the omitted information was so important)
- framing feedback in a respectful manner (the importance of this teaching strategy is in line with results from Kluger and DeNisi, showing that feedback, that threatens self-esteem is going to be denied [15])
- using questions to promote clinical thinking

Eppich et al. recommend that simulation is an ideal way to teach telephone communication between physicians [14]. Although this study focused on telephone conversations among physicians, the conversations described in this article share essential features with the communication between patients and doctors, suggesting that simulation is also a valuable way to teach telephone consultations between doctors and patients. That telephone consultation can be successfully taught with simulation is supported by another qualitative study, which investigated the experiences of general practitioner trainees in the UK, with respect to telephone consultations with patients. The trainees in this study stressed the importance of experience in and training of telephone consultations. Positive experiences of the trainees with telephone conversations were correlated with the level of supervision and feedback they received [16]. Practice, gaining experience and feedback are key elements of teaching and learning with simulation.

In Switzerland, the catalog of learning objectives for undergraduate medical training, known as PROFILES (Principle and Relevant Objectives and Framework for Integrative Learning and Education in Switzerland), includes learning objectives related to telephone consultations. One of the objectives is to be able to answer patients’ questions via telephone in a competent way [17]. Another objective is to decide whether self-treatment of the patient at home is sufficient or if treatment by a specialist in a medical facility is necessary and to advice the patient accordingly [17]. 

At the medical faculty of the University of Bern, we established a designated course in “Telephone Consultation for Medical Emergencies” in 2012, which is taught to 5^th^ year students. 

## 2. Project description

The didactic concept (described in paragraph 2.2.) and the content of the course were developed by an interdisciplinary group of experts in the Medical Faculty’s Committee for Communication Skills. The committee oversees the longitudinal curriculum in communication skills in the medical Bachelor/Master program in Bern. 

### 2.1. Course preparation

#### 2.1.1. Student materials

Based on quality criteria for telephone consultations outlined in the introduction, the guidelines were oriented towards the RICE criteria [8]. A list of signals that should prompt the physician to initiate an in-person visit (“red flags”) was compiled [18] and a guideline on how to document the call was prepared. General communication skills needed in telephone consultations along with requirements specific to this way of communication were gathered. A script combining the theoretical foundations with organizational course details was created and distributed to the students for self-study prior to the course. 

##### 2.1.2. Scenarios for simulation 

Scenarios were developed collaboratively by clinical experts and simulation specialists. Typical symptoms prompting patients to call a doctor were selected as topics of the cases/scenarios. A case writing template was created by simulations experts and provided to clinicians, who filled in the template, creating simulation scenarios that were reviewed and finalized by a simulation expert in cooperation with the clinician author. 

A pool of scenarios was developed, encompassing epicondylitis, knee trauma, obstipation, diarrhea, exanthema, pharyngitis, and a parent calling about their child suffering from fever, or a child suffering from ear pain.

Each year, four of these scenarios are selected to be taught. We ensure that each pair of students is assigned at least one scenario involving a parent calling on behalf of a child, representing an even higher level of indirect communication than “regular” telephone consultation. 

##### 2.1.3. Checklists

A general checklist based on the RICE criteria for telephone consultations was developed and distributed to the students for preparation of the course and the simulation. 

Based on this general checklist, case-specific checklists for each scenario were created to be used during the course (see details paragraph 2.2.). The main difference between the general checklist and the case-specific checklists is the inclusion of case-specific “red-flags”. As mentioned above, “red flags” are symptoms and/or signs that should prompt a physician to initiate in person treatment of the patient e.g. neurological deficits in a patient with lumbar pain. An example of a case-specific checklist is shown in attachment 1 . 

##### 2.1.4. Simulated patients (SP)

SPs were selected from an internal data base by the following criteria: quality of performance, ability to give feedback and availability. Criteria that are usually important in casting SPs for specific roles like age, body mass index, scars were not important in recruitment of SPs for telephone consultations, since they are not observable in telephone consultations. 

According to the Association of Standardized Patient Educators (ASPE) Standards of Best Practice in working with SPs (SOBP) [19], SPs were trained for the simulation combining interactive discussion of theoretical background and deliberate practice [20]. 

Besides performing the patient’s role during the simulation, SPs were trained in giving feedback. Feedback training is based on a combination of different theories, including:

- positive framing of feedback [21],
- the “Pendelton model”: self-reflection, feedback on areas that went well as well as on areas for improvement, action plan and positive wrap up [22],
- Gibbs’ “Witness Model” in which the person offering feedback describes specific observable behavior and the impact it had on him or her [23].

SPs are not supposed to focus on medical content in their feedback. Feedback on medical content is provided by peers (see details in paragraph 2.2.). 

The SPs feedback is focused on the patient`s perspective, including their perception of the use of understandable language and the clarity of information provided regarding the explanation of symptoms and further management. Other important elements of SP feedback are their responses to the emotional aspect of the consultation, like the feeling of empathy they might experience from a student. 

#### 2.2. Didactic concept

**Figure 1:**
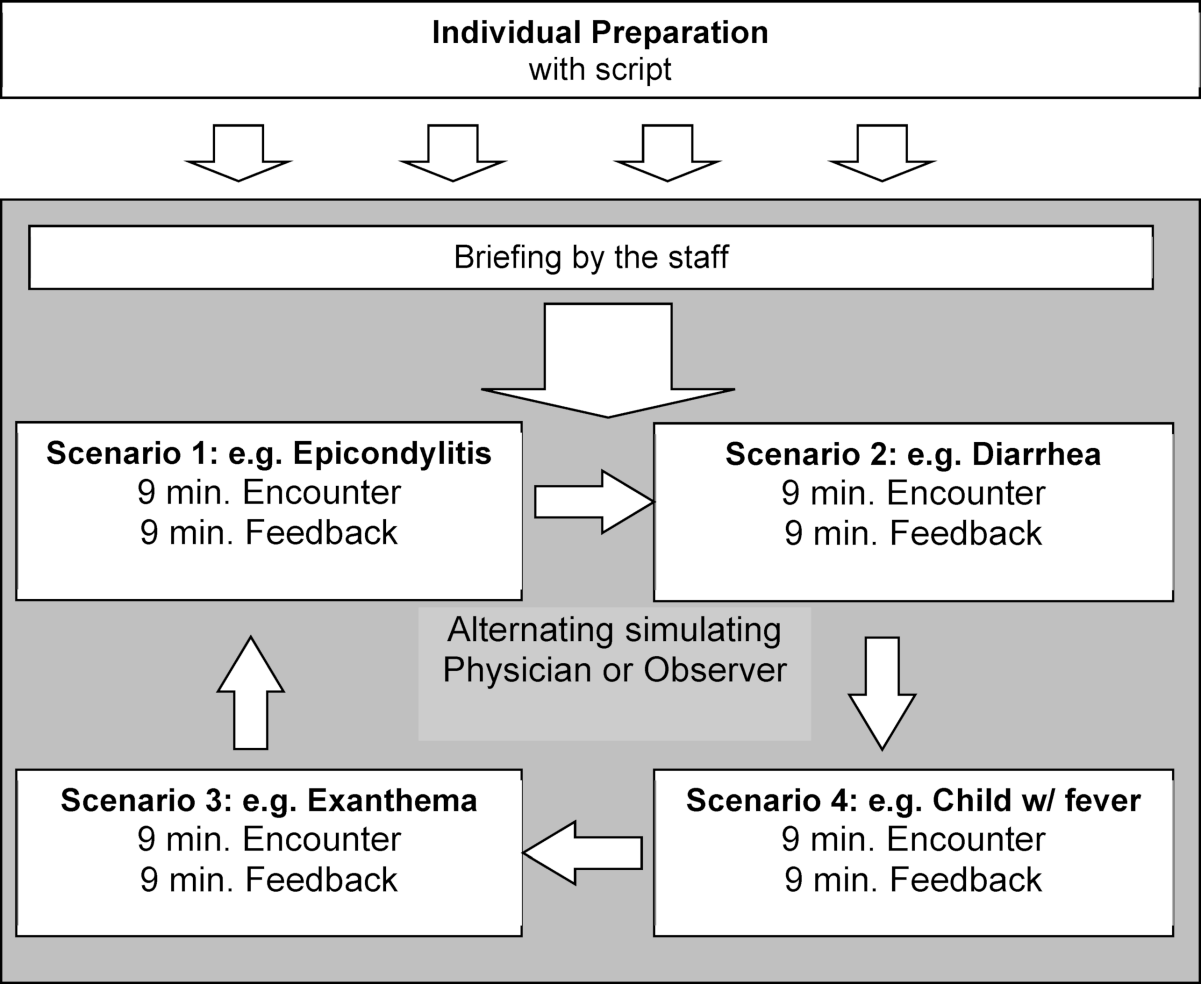
Course format

The necessary theoretical foundation for communication via telephone is delivered to the students in form of a written summary of the basic concepts, which students prepare in self-study. Following this preparation, students come to the simulation center in groups of 8, rotating in pairs through 4 scenarios with SPs (see figure 1 (Fig. 1)). Each student takes the role of the physician in 2 of the scenarios and observes 2 consultations of his/her peer with other SPs. 

Upon arrival at the simulation center, students are oriented by the staff about their tasks for approximately 10 minutes. Afterwards, the 4 students taking the role of a physician wait in front of 4 rooms. The 4 observing peer students stay next to their respective partners during the interview and are handed the case-specific checklists. The 4 SPs wait in separate rooms. All rooms are equipped with a telephone. Upon a common signal, the students enter their rooms and receive a telephone call from the SPs. Each student in the physician’s role picks up the phone and starts the conversation, while their observing peer fills in the checklist.

**Figure 2:**
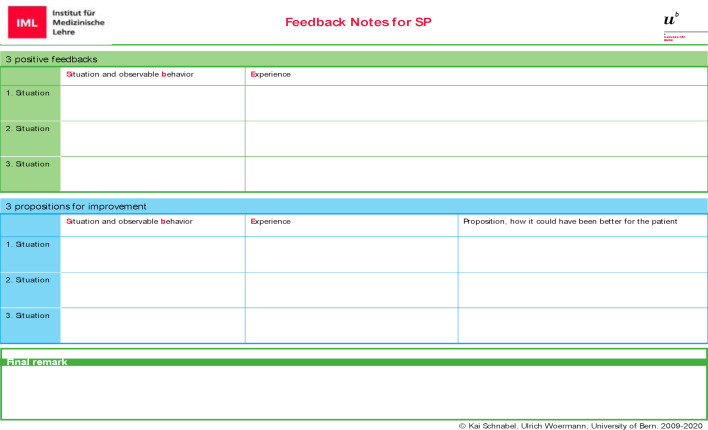
Feedback Template

Each telephone encounters lasts up to 9 minutes. When the conversation is finished, the SPs write down their feedback on provided feedback sheets (see figure 2 (Fig. 2)). During this time, the student pairs stay in their simulation rooms and exchange their experiences including the peer-feedback, based on the case-specific checklist. Once the SP’s written comments are completed, he/she joins the students and provides oral feedback based on the notes on the feedback sheet. In the end, the feedback-sheet with the notes is handed to the student. This process also takes around 9 minutes. 

Following this sequence, 2 minutes are reserved for re-organization and rotation. Students rotate from one case to the next, changing their roles of actively simulating and observing. SPs walk back to their rooms preparing to play their role with the next pair of students. During the training, time is kept by a central gong system. 

The rotation ends once the student pairs have completed all 4 cases. While the students go on to meet a clinical expert to debrief for 30 minutes, SPs restart with the next group of students after a 10 minutes break. This way, a total of 24 students (3 x 8) can complete the course in one afternoon. 

#### 2.3. Evaluation 

We have been successfully teaching the course since 2012. The course has been evaluated with different strategies:

- Student survey
- Collection and analysis of students comments and remarks during the debriefing sessions

While the student survey was done only once so far, the evaluation through students’ comments during the debriefing is an ongoing process. The evaluations have demonstrated the students’ high acceptance of the course format. As examples we present here the results of the survey done in 2014 and the analysis of the students` comments collected during debriefings in 2019. 

##### 2.3.1. Survey 

In 2014 all students taking part in the training were asked to rate 5 statements describing their experience during the course using the following rating scale: 1=agree completely, 2=tend to agree, 3=partly agree and partly disagree, 4=tend to disagree and 5=do not agree at all. In addition to rating the statements, students were asked to comment on the training and give suggestions for improvement. The survey was handed to the students in writing, with checkboxes to mark the ratings 1-5 and additional space for individual comments. A total of 179 of 185 students completed the survey.

The result of the rating expressed in mean value ± standard deviation was:

- “I have learned a lot of new skills” (1,6±0,6).
- “I consider what I learned in this course as important for my future job” (1,1±0,4).
- “The feedback of the SPs was helpful for me” (1,4±0,6).
- “Observing an encounter and filling in a checklist added to the learning” (1,5±0,7).
- “I wish, I could have practiced on all 4 scenarios (double training time)” (2,5±1,2).

In their comments, the students mostly complimented the SPs on their role-play and feedback. They stated that, they would like to have more training opportunities of this kind. A point of criticism mentioned was that the observing student could only hear, his/her colleague in the simulation, but not the SP, since the SP was on the phone in another room. Thus, it was difficult for the observer to follow the conversation and fill in the checklist. As a result of this feedback, telephones with loudspeakers were purchased, allowing the observing student to hear both, his/her colleague and the SP. 

##### 2.3.2. Debriefing 

Once a group of 8 students has completed 4 scenarios, they meet with a clinical expert for debriefing and course evaluation. In 2019 the feedback from 10 groups of students (corresponding to 80 students total) was documented by the clinical experts during the debriefing. The experts wrote the students’ comments in textboxes of a questionnaire provided on a tablet computer with a commercially available software (Survey Gizmo [https://www.alchemer.com/]).

In summary the students, stated with regard to:

- the scenarios
  - 3 out of 4 scenarios were described as being realistic and relevant; one scenario had a mixed reaction (details below)
  - some scenarios were more difficult than others, the level of difficulty of the scenarios was just right.
  - no suggestions for improvement of the scenarios, the only improvement discussed was, to exchange the one scenario mentioned above for another one.
- the SPs
  - the performance during the simulation was good and authentic
  - the feedback was helpful in general, most of the time the feedback was authentic and constructive, could be more critical sometimes
- the documents for preparation and the organization
  - adequate documents, good organization
- the most instructive aspect of the training
  - opportunity to practice
  - getting feedback
  - experiencing a patient interview without seeing the patient
- suggestions for improvement
  - not knowing the diagnosis and case-specific checklist ahead of time during the preparation
  - more cases with positive red-flags to add more difficulty

As mentioned above one scenario of the course had a mixed reaction. The scenario describes an epicondylitis. Some students offered the criticism that it would be unlikely that patients would call a doctor if they had this chronic condition. Many students had difficulties making the correct diagnosis because they missed the elbow’s redness and swelling, which was not visible over the phone. However, other students had the realization that the point of including these kinds of scenarios in this training was to lead them to actively inquire about symptoms that might be invisible to the physician.

The students’ suggestion to receive the telephone call in the simulation without knowing the diagnosis and the case-specific checklist ahead of time refers to an organizational misfortune, in which the students were informed of the diagnosis in their cases during the preparation time. The cause of the incident was identified and taken care of. 

#### 2.4. Assessment

The course’s learning objectives are not assessed in a designated exam. The skills required for telephone consultations are part of the pool of learning objectives that are assessed in the OSCE following the 5^th^ year of studies and the clinical skills assessment of the Federal Licensing Exam. OSCE cases simulating a telephone consultation have been used in exams in the past and will be randomly used again. 

Performance data of the students were not collected for research and lack a control group.

Data integrity and safety prevent the publication of these OSCE results. 

## 3. Discussion

The strength of the designed course lies in specifically addressing evidence-based differences between face-to-face interactions and telephone consultations:

The requirement for high verbal cue sensitivity [7] is addressed by incorporating cues in the scenario. SPs are trained to express these cues, e.g. a mother calling on behalf of her child suffering from fever is very worried and will express this feeling through the tone of her voice. The students are expected to pick up on this cue. The SPs include in their feedback the patient’s perspective regarding the way this feeling was addressed by the student. Feedback is known to be a very powerful tool for learning [23], [24] and was mentioned by the students during the debriefing as being one of the most instructive aspects of the training. 

The need for focused history taking due to the lack of visual cues [6] is practiced through scenarios, based on a diagnosis that can be missed, when visual cues cannot be seen. The epicondylitis scenario is an example of this kind of scenario. The diagnosis was missed frequently by the students. Students doubted that patients with epicondylitis would contact a doctor via phone, questioning the authenticity of the scenario. However, the authors (and many students, as evidenced by their feedback in the debrief) feel that this scenario is especially valuable because it demonstrates the critical importance of precise questions for certain symptoms in the absence of visual cues. It is important for the clinical expert to debrief this special feature of telephone consultations. 

Focused history taking [7] is also supported by rising the awareness for case-specific “red-flags”. These “red-flags” are outlined in the script for course preparation, included case-specific checklists that are discussed in peer-feedback and are discussed again in the debriefing with clinical experts. 

By constructing the checklists according to the RICE criteria, evidence from the literature supports the validity of teaching this concept [9]. Research by Eppich et al. as well as Chaudhry et al. supports the effectiveness of simulation in teaching telephone consultation [14], [16].

In simulation, the transfer of knowledge to the clinical practice is often discussed [25]. Since the scenarios, script and course concept were developed in close co-operation of simulation and clinical experts, clinical relevance of the course is granted. This is confirmed by the feedback of the students in the survey and in the debriefing. One problem for the transfer of the skills acquired in the simulation center to the clinical practice is, the fact, that the 5th year students are not in regular clinical practice. Thus, they do not have the opportunity to apply the obtained skills right away. 

Building on evidence from the literature, we have implemented a telephone consultations course that is well received by the students. Data from a clinical skills assessment (e.g. OSCE) testing the performance of the students would strengthen the evidence for the course, but are currently not available in publishable form. 

## 4. Conclusion & future perspectives

Telephone consultations differ from face-to-face interaction and involve skills that require specific training. Simulation is a method that is highly accepted by the students, is supported by evidence in the literature and can be carried out reliably. The importance of telephone consultation is increasing. Telephone consultation is currently greatly encouraged due to the COVID-19 pandemic, causing government institutions to ask patients to contact healthcare professionals via telephone before attending a medical faculty [5]. The national state of emergency with impending curfew creates a new momentum for the promotion of advanced skills in telemedicine. In the clinic different means of telecommunication are promoted. At the same time, forced by the suspension of all on-site instruction and simulation for the protection of SPs and learners alike, medical educators are in the process of developing formats for remote teaching and learning as we write. 

Another aspect to be considered in the future is that remote communication will not be restricted to the transmission of acoustic signals forever. Smartphones and computers equipped with cameras for photo and film already broaden the possibilities for tele-consultations and should be considered for inclusion in future trainings. 

## Acknowledgements

We would like to thank the Committee for Communication Skills at the Medical Faculty of the University of Bern, especially Mireille Schaufelberger, Adrian Göldlin and Regina Ahrens for their contributions to the development of the course. We would like to thank Daniel Bauer for his assistance in reviewing the script and last but not least Cathy Smith for her review of the script.

## Profile

**Name of school: **University of Bern, Bern, Switzerland

**Study program/occupation: **Medical Faculty

**Number of students per year and/or per semester: **ca. 340 students / year (since 2018)

**Has a longitudinal curriculum covering communication been implemented? **Yes

**At which semester levels are communicative and social competencies taught?**

- Taking a medical history & providing/receiving feedback – 1^st^ year
- Formative OSCE; including SP feedback on communication – 1^st^ year
- Formative OSCE; including SP feedback on communication – 3^rd^ year
- Geriatric assessment – 4^th^ year
- Clinical skills training (incl. learning objectives with regard to communication) – 4^th^ year
- Communication in anesthesiology – 4^th^ year
- Challenging conversations, part 1 – 4^th^ year
- Telephone consultation – 5^th^ year
- Formative OSCE; including SP feedback on communication – 6^th^ year
- Challenging conversations, part 2 – 6^th^ year

**Which teaching formats are used? **Mainly small groups tutorials engaging students in simulation with simulated participants

**During which semesters are communicative and social competencies tested (formative, pass/fail, graded)?**

- Formative assessments in the 1^st^, 3^rd^ and 6^th^ year
- Summative (pass/fail) assessments in the 3^rd^, 5^th^ and after the 6^th^ year (Federal licensing exam)

**Which assessment formats are used? **OCSEs with 4 (formative) or 10-12 (summative) cases of 8-13 minutes

**Who (e.g. hospital, institution) is in charge of development and implementation? **The University of Bern’s Medical Faculty installed a Committee for Communication Skills in charge of the design and implementation of the communication skills curriculum. The committee consists of an interdisciplinary group of clinical and didactic experts, coordinated by the Institute of Primary Health Care and the Institute for Medical Education.

## Current professional roles of the authors

- Beate Brem works as scientific collaborator at the Department for Education and Media of the Institute for Medical Education, University of Bern in Switzerland. She is head of the Simulated Patient (SP) program.
- Kai Schnabel is head of the department for Education and Media of the Institute for Medical Education, University of Bern in Switzerland.
- Ulrich Woermann works as scientific collaborator at the Department for Education and Media of the Institute for Medical Education, University of Bern, Switzerland. He is head of the e-Learning unit.
- Roman Hari is head of the department of education at the Institute of Primary Healthcare, University of Bern, Switzerland. He also works as a general practitioner in Burgdorf, Switzerland.
- Anina Pless works as a scientific collaborator in the Department of Education at the Institute of Primary Healthcare of Bern, Switzerland, where she is responsible for the field “teaching communication”. She also works at the medical policlinic of the University Hospital of Zurich, Switzerland.

## Competing interests

The authors declare that they have no competing interests. 

## Supplementary Material

Example of a Checklist for Peer-Feedback, Scenario “Child with Fever” (authors’ translation)
