# Supplementary material for: “Telephone consultation for medical emergencies” – development, implementation and evaluation of a course in identifying and handling medical emergencies via telephone for 5th year medical students
Source: GMS J Med Educ. 2021 Mar 15;38(3):Doc63. doi: 10.3205/zma001459 (PMC7994869; doi:10.3205/zma001459)
Supplement: Example of a Checklist for Peer-Feedback, Scenario “Child with Fever” (authors’ translation) [file JME-38-3-63-s-001.pdf]

Attachment 1: Example of a Checklist for Peer-Feedback, Scenario „Child with Fever“ (authors` translation)

	Not inquired	Partly inquired	Completely inquired
Introduces himself/herself incl. name and position *(Example: resident on call, pediatrician, etc.)*	O	O	O
Asks for name, age and telephone number	O	O	O
Starts with open question (*Example: How can I help you?)*	O	O	O
Explores presenting complaint	O	O	O
• Duration of symptoms	O	O	O
• Associated symptoms	O	O	O
o Ear or throat pain	O	O	O
o Headaches	O	O	O
o Cough	O	O	O
o Abdominal pain, vomiting, diarrhea	O	O	O
o Exanthema	O	O	O
o Pain in joints	O	O	O
• Urine (amount, color, smell)	O	O	O
• Degree of disturbance (general condition)	O	O	O
• Vaccinations	O	O	O
• Pre-existing conditions incl. childhood diseases	O	O	O
• Sick contacts	O	O	O
• Measures taken so far	O	O	O
„Red Flags“	O	O	O
• Fever › 39,5 °C	O	O	O
• Difficulty breathing	O	O	O
• Headache (stiff neck)	O	O	O
• Level of consciousness	O	O	O
• Rashes / exanthema (i.e. purpura, etc.)	O	O	O
• Seizures	O	O	O
• Recent travels abroad	O	O	O
• Easily dazzled, shade loving	O	O	O
• Sensitivity to touch	O	O	O
Asks for medical history in General	O	O	O
Asks for medication	O	O	O
Questions are easy to understand and are asked one by one without overwhelming the patient or parent, respectively)	O	O	O
Summarizes the problem and asks for confirmation of correctness	O	O	O
Asks for expectations	O	O	O
	**Correct**		**Incorrect**
Establishes a management plan	O		O
Checks, if management plan is realistic	O		O
→ Treatment at home	O		O

Attachment 1 to: Brem BG, Schnabel KP, Woermann U, Hari R, Pless A. *“Telephone consultation for medical emergencies” – development, implementation and evaluation of a course in identifying and handling medical emergencies via telephone for 5th medical students.* GMS J Med Educ. 2021;38(3):Doc63. DOI: 10.3205/zma001459

Explains	O	O	O
• What to do, if problem exacerbates	O	O	O
• When to call again	O	O	O
o Body temperature continues rising	O	O	O
o General condition worsens	O	O	O
o Headache	O	O	O
o Difficulty breathing	O	O	O
o Exanthema	O	O	O
	**Not inquired**	**Partly inquired**	**Completely inquired**
Asks the caller`s consent to management plan	O	O	O
Asks the caller to repeat the management plan	O	O	O
Asks for lack of clarity or further questions	O	O	O
Concludes conversation	O	O	O
